# Perceptions among transgender women of factors associated with the access to HIV/AIDS-related health services in Yogyakarta, Indonesia

**DOI:** 10.1371/journal.pone.0221013

**Published:** 2019-08-15

**Authors:** Nelsensius Klau Fauk, Maria Silvia Merry, Sukma Putra, Mitra Andhini Sigilipoe, Rik Crutzen, Lillian Mwanri

**Affiliations:** 1 Institute of Resource Governance and Social Change, Kupang, Nusa Tenggara Timur, Indonesia; 2 College of Medicine and Public Health, Flinders University, Adelaide, South Australia, Australia; 3 Medicine Faculty, Duta Wacana Christian University, Yogyakarta, Indonesia; 4 Bina Nusantara University International, Senayan, Jakarta, Indonesia; 5 Department of Health Promotion, Maastricht University, Maastricht, The Netherlands; National University of Singapore, SINGAPORE

## Abstract

Access to HIV/AIDS-related health services among transgender women living with HIV is still a major public health issue in many developing countries, and Indonesia is not an exception. However, reportedly compared to other settings in the country, transgender women in Yogyakarta have a good access to the HIV-related health services. This study aimed to explore perceptions among transgender women living with HIV, locally known as *waria*, of factors supportive of their access to the services in Yogyakarta, Indonesia. A qualitative inquiry using in-depth interview method was conducted from December 2017 to February 2018 to collect the data from a selection of *waria* living with HIV (n = 29) recruited using both purposive and snowball sampling techniques. Data analysis employed a thematic approach which was guided by the framework analysis for qualitative data. The findings indicated several health service system-related determinants supportive of *waria’s* access to HIV/AIDS-related health services. These included the availability of the services, the simplicity and convenience of accessibility to the services and the comfort felt by the participants while accessing the services. Health professionals’ positive attitudes during healthcare provision, social relationships between *waria* and health professionals, proximity to healthcare facilities, free access to the services, and information sessions on HIV infection and prevention were also reported to enable participants’ access to the services. These findings call to efforts and strengthening of HIV health service system to support and provide equal access to HIV/AIDS-related services including to all Indonesians living with HIV, but more so for transgender women and other high-risk groups such as sex workers and their clients and men who have sex with men.

## Introduction

Access to HIV/AIDS-related health services including voluntary counselling and testing (VCT) of individuals at risk for HIV infection and antiretroviral therapy (ART) among those living with HIV, has been considered as a key strategy for HIV prevention and AIDS-related death reduction [1–3], and many countries across the world have supported the provision of HIV-related health services to control HIV among different vulnerable populations. In Indonesia, there is limited availability and inequitable distribution of resources which act as a hinderance to effective delivery of HIV/AIDS healthcare services to population groups within and between societies in the country [4–7]. Furthermore, evidence exists of low accessibility to HIV/AIDS healthcare services especially ART among high-risk groups, including sex workers and their clients, men who have sex with men, and transgender persons [8–13]. In addition, Indonesia has recently experienced a significant proportional increase of AIDS-related deaths, AIDS cases and HIV cases, corresponding to 68%, 161% and 316% respectively during the period from 2010 to 2016, contrary to the global reduction of 32% of AIDS-related deaths and 16% of HIV infections during the same period [14, 15].

Transgender women, commonly known in Indonesia as *waria*, are one of the highly susceptible groups to HIV infections due to their engagement in unprotected anal intercourse (UAI) with multiple sex partners [16, 17]. It is also recognised that transgender women especially those who are sex workers are at heightened risk of HIV infections due to structural, interpersonal, and individual vulnerabilities [18, 19]. Of an estimated two million *waria* populations in the country, 24.8% reported to be living with HIV infection in 2016 [13, 14]. It is acknowledged that transgender women and other vulnerable populations experience high levels of HIV stigma and other social and structural factors which act as important barriers for them to access HIV care services [20].

However, studies elsewhere have reported knowledge or information about HIV/AIDS healthcare services and availability of these services as factors supportive to accessing the services [21–24]. Proximity to healthcare facilities and affordability including of medical and transport costs to healthcare facilities have also been reported to enable transgender women’s access to HIV/AIDS healthcare services [25, 26]. Furthermore, different types of support received from their sex partners and friends have been stated as factors motivating them to utilise the HIV/AIDS healthcare services [26, 27].

Addressing the HIV epidemic, especially among vulnerable populations in Indonesia would be supported by a health system that responds and supports the access to HIV/AIDS-related health services by different vulnerable populations. An understanding of facilitators needed to improve access to HIV/AIDS-related health service system for *waria* is also critical in targeting this very vulnerable group. It has been reported that access to HIV-related health services in Indonesia is difficult for people living with HIV due to various reasons [28–32]. However, compared to other settings in Indonesia, Yogyakarta is a place where the services are well supported in healthcare facilities and easily accessible to people living with HIV including transgender women [33, 34]. However, evidence on factors supportive of accessibility to the services among people living with HIV is still scarce in Indonesia. Guided by the access to healthcare conceptual framework, this study aimed to explore the perceptions among transgender women of supporting factors for accessibility to HIV/AIDS-related health services in Yogyakarta, Indonesia. Understanding enabling factors for the access to the services among *waria* or transgender women in this setting can be useful information for HIV-related healthcare providers, governmental and non-governmental organisations and institutions concerned with HIV/AIDS issue to improve the access of people with HIV to the services in other settings in the country.

## Methods

Consolidated criteria for reporting qualitative studies (COREQ) checklist [35] was used to guide the report of the methods section of this study. The COREQ checklist contains 32 (S1 Fig) required items for explicit and comprehensive reporting of qualitative studies especially interviews and focus groups [35].

### Theoretical framework

The access to healthcare conceptual framework [36] guided the conceptualisation and further the discussion of the study findings. The five dimensions of accessibility offered by this framework (approachability, acceptability, availability, affordability, and appropriateness) and further five corresponding abilities (ability to perceive, ability to seek, ability to reach, ability to pay and ability to engage) helped our theorising of the interactions between populations and service against these dimensions to generate access [36, 37] (see Table 1). Access to healthcare services can be achieved if the five accessibility dimensions of supply side are supported by the five corresponding abilities of demand side or individuals facing health needs. The interaction between the dimensions and abilities is presented below using the context of *waria* who participated in this study. We espoused the framework in the context of *waria* in Yogyakarta, and suggest that in order to be accessible, health services should be made *approachable* to *waria*. *Approachability* would mean that the services should be well known to *waria* and the *waria* should have *ability to perceive* the need for the services (based on their knowledge about the services). The accessibility to health services would also be influenced by whether or not the services are *acceptable* to *waria*. *Acceptability* may refer to the cultural and social aspects that determine *waria’s* acceptance of care, judged by their perceptions of the appropriateness of that care. *Acceptability* of health services would also be determined by *waria’*s *ability to seek* which could be influenced by their personal values or knowledge of existence of the services. The *availability* of services (i.e. the existence of HIV/AIDS-related healthcare services) for *waria* and *waria’*s *ability to reach* the services are aspects supportive of the accessibility of health services. *Waria’*s *ability to reach* could be influenced by their knowledge about the existence of health services and procedure to access them, as well as availability of transport to go to healthcare facilities. Additionally, *accessibility* of services would also be determined by the *affordability* or the economic capacity of *waria* to spend resources and time to access the services. This *affordability* dimension would also be supported by *waria’*s *ability to pay* for transport and medical costs, and to allocate time to use the services. Finally, *appropriateness* of the HIV/AIDS-related health services would refer to whether the services meet *waria’*s needs, including whether *waria* trusted the professionals or care providers, additional dimension that supports the accessibility to services. This dimension would also be determined by *waria’*s *ability to engage* in the services. Such abilities would be influenced by individual *waria’*s capacity and motivation to utilise the health services.

**Table 1:** Access to healthcare framework.

Concept	Definition
*Five dimensions of accessibility (Supply side)*	
Approachability	refers to the fact that the existing health services can be identified and reached by people facing health needs
Availability	relates to the physical existence of health services
Affordability	reflects the prices of health services and capacity of people to spend resources and time on the services
Acceptability	refers to whether or not health services provided for people are culturally and socially acceptable
Appropriateness	refers to the extent to which the health services serve the needs of the people
*Five corresponding abilities (Demand side)*	
Ability to perceive	refers to people’s knowledge about health and health services available for them
Ability to seek	refers to individual capacity to choose to seek health services.
Ability to reach	refers to the ability of individuals facing health needs to have the knowledge of health services and physical capacity (e.g., transport for mobility) to reach healthcare facilities where the health services are available.
Ability to pay	refers to economic capacity of each individual to pay for healthcare services, and
Ability to engage	relates to the participation of individuals in decision making about the access to health services

Source: adapted from Levesque et al [36].

### Procedure and data collection

A qualitative inquiry with transgender women or *waria* populations living with HIV was conducted from December 2017 to February 2018 in Yogyakarta, Indonesia. The study participants (n = 29) were recruited purposively and by using snowball sampling technique. As the potential participants are one of vulnerable groups and therefore hard to reach, the identification of a focal personal who was known to *waria’s* social networks followed by the use of snowball technique was considered an effective mean of recruiting participants. To begin recruitment process, the field researchers made initial contact to meet with the director of a known non-governmental organisation (NGO) that provides supports for *waria* populations in Yogyakarta. The director assisted to distribute the information sheet containing the information about the study and contact details of field researchers to potential participants who might be willing to take part in this study. Several initial participants were also asked to spread the study information sheet to other potential participants in a snowball fashion. This process was recursive until the data saturation was reached as the last few participants provided information similar to that of previous participants. Of 32 potential participants who contacted us three withdrew their participation due to personal reasons. This recruitment technique provided sufficient sample of participants for data collection, but they were all from the same social network of people who knew each other. The recruitment of the participants was based on several inclusion criteria including: (i) one who was self-identifying as *waria*, (ii) self-reporting to be HIV positive, (iii) 18 years old or above, and (iv) had accessed the HIV/AIDS-related healthcare services.

One-to-one in-depth interviews were conducted by two field researchers. The locations where the interviews took place were the participants’ houses and a private room at the shelter for *waria*, which belonged to the NGO. These locations were identified based on preferences by the participants. Nobody other than the researcher and the participant was present during the interviews and the range duration of each interview was 45 to 90 minutes. The field researchers did not know any of the participants prior to this study. Topics (S2 Fig) that were focused on during the interviews included access to HIV/AIDS-related health services, HIV/AIDS-related health service information, experiences related to the service delivery at healthcare facilities, procedure to access the services, the attitudes of health professionals, *waria’s* interactions and social relationships with health professionals, and affordability to access the service. At the end of the interviews, each participant was asked whether they would like to see a copy of interview transcript and edit it prior to us analysing it. However, none of the participants required to see it. No repeated interview with any of the participants was conducted. Interviews were conducted in Bahasa.

Before commencing the interviews, participants were again informed about the purpose of the study. They were advised about the voluntary nature of their participation and that they remained their rights to withdraw from this study if they feel uncomfortable with the topics being asked without any consequences. At the beginning they were informed about the duration of the interview which would take approximately 45 to 90 minutes and that the interview would be recorded using a tape-recorder and that the interviewer would also take notes during the interview. They were also assured that to prevent the possibility to linking back the data or information to each individual in the future, each data item would be made confidential and anonymous by assigning a unique Study Identification Number (R1, R2, …). Prior to the interviews, participants were informed that ethics approval for this study was obtained from Medicine Research Ethics Committee, Duta Wacana Christian University, Indonesia (ref: 558/C.16/FK/2017). The participants signed and returned a written informed consent at the interview day.

### Data analysis

The first two authors who are fluent in both Bahasa and English transcribed the recorded data verbatim into coding sheet and translated into English. Cross check and comparison of the transcription and translation between the two authors to maintain the quality and validity of the data took place during the transcription and translation process. Data were further checked for clarity of transcription and accuracy of translation by other authors. Data management and analysis were conducted manually. Guided by step by step framework analysis for qualitative data by Braun and Clarke [38], data were thematically analysed as follows:

1. *Familiarisation* with the transcripts through reading the transcripts repeatedly, marking ideas and giving comments to search for meanings, patterns and ideas; and *generation of initial codes* to data extracts from individual transcripts by writing notes on the texts being analysed. This was followed by close coding which led to the collection of a manageable number of codes.
2. The *search for themes* was performed through sorting different codes into potential themes and sub-themes. The same themes or sub-themes were grouped together, and all the relevant coded data extracts were collated within the identified themes and sub-themes. This process led to the collection of candidate themes and sub-themes and the extracts of data that have been coded in relation to them.
3. The *review and refinement of the candidate themes and sub-themes*. All the collated extracts for each theme were read to see whether or not they appeared to form a coherent pattern. This was to ensure that the themes were really themes because supported by sufficient data, if not then discarded, and to see whether the themes could collapse into each other or could be broken down to separate themes.
4. *Defining and naming the themes* through the identification of the essences of what each theme was about (as well as themes overall) and the determination of what aspect of the data each theme captured. This was performed by going back to the collated data extracts for each theme and organising them into a coherent and internally consistent account, with accompanying narrative.

## Results

### Characteristics of the participants

Participants’ age ranged from 32 to 57 years, with the mean being 44 years. Participants were categorised into three different age groups and originally from eight different provinces in Indonesia (see Table 2). The education backgrounds of the study participant varied. All the participants had sex work as one of their sources of income and also had part time jobs. All the participants are living with HIV. Several of them had also been infected with other sexually transmitted infections such as syphilis, gonorrhoea, and genital warts. Four participants had also been diagnosed with tuberculosis. All the participants were on ART.

**Table 2:** Characteristics of the participants.

Characteristics	No. of Respondents N = 29 (%)
*Age*	
30–39	9 (31)
40–49	11 (38)
50–59	9 (31)
*HIV diagnosis*	
1 to 5 years ago	9 (31)
6 to 10 years ago	13 (45)
11 to 15 years ago	7 (24)
*Province of origin*	
Special Region of Yogyakarta	11 (38)
Central Java	6 (21)
West Java	4 (14)
North Sumatera	2 (7)
South Sumatera	2 (7)
East Java	2 (7)
Riau Islands	1 (3)
Bengkulu	1 (3)
*Education*	
Senior High school graduates	9 (31)
Junior High school graduates	9 (31)
Elementary school graduates	6 (21)
Elementary school drop outs	5 (17)
*Occupation*	
Makeup stylists	8 (28)
NGO workers (volunteers)	6 (21)
Food stalls’ assistants	5 (17)
Street singers (*ngamen*)	4 (14)
*Angkringan*, Chicken, and Coconuts sellers	3 (10)
Housekeeper, online motor-taxi driver and staff at a spa company	3 (10)

Findings were grouped into four main themes including (i) availability and appropriateness of health services, (ii) acceptability of HIV-related health services and the access procedures, (iii) approachability of HIV-related health services (iv) health professionals’ approachability and impact of their positive attitude, and (v) affordability of services and proximity to healthcare facilities. The selection of the themes was also guided by the constructs of access to healthcare framework to reflect a logical pattern of how different aspects of health service system play supportive roles in facilitating accessibility to HIV-related health services among transgender women living with HIV.

### Availability and appropriateness of HIV-related health services

The HIV/AIDS-related health services and HIV-related information were reported to be available and accessible to *waria* populations. All the participants accessed services such as HIV counselling, viral load and CD4 check, STIs (sexually transmitted infections) test, antiretroviral (ARV), blood sugar, uric acid and cholesterol test, and nutritional foods:

*“Health services are available for us [waria]*. *At first*, *we [waria] usually do STIs test other than HIV and if one is infected then she will be recommended to attend VCT*, *and if one is HIV positive then she will be recommended to do other test such as CD4 test or viral load check*, *and access ARV*”

*(R12*: *34 years old).*

*“The [HIV-related] services are available for all HIV-positive people*. *I take my medicine [ARV] every month and do the counselling every three months”*

*(R4*: *39 years old).*

*“I access the services regularly*, *I also check blood sugar*, *uric acid*, *cholesterol*, *CD4”*

*(R19*: *45 years old).*

The services from community health centre and/or hospital seemed to be appropriate and easy for the study participants to access them. The convenience and the contentment experiences and the perceived need of the participants for the services were articulated and seemed to be added determinants to accessing HIV-related health services:

*“I feel comfortable every time I access the [HIV/AIDS] health services*. *I think they [nurses and doctors] know us [waria] and support us to access the services*. *I regularly undergo medical check-up and access medicine up to now”*

*(R11*: *33 years old).*

*“Health services I need are mainly ARV*, *CD4 test and counselling*. *These services are available in some hospitals and community health centres and free for us [HIV patients]*, *that is why I am on ART up to now*. *…*. *The doctor and nurse whom I met in the hospital encourage me to continue treatment*. *I listen to them and feel motivated by what they said”*

*(R2*: *51 years old).*

*“…*. *I utilise the [HIV-related health] services because I need them*, *especially ARV and CD4 test*. *Nurses and doctors who handle us [HIV-positive Waria] also remind me to adhere to the treatment so that I can become healthier*. *I trust them and what they said”*

*(R14*: *41 years old).*

### Acceptability of HIV-related health services and the access procedures

Participants’ satisfaction with the available services, acceptability of the health services and the service providers and the way services were provided were mentioned as part of factors determining accessibility of services by *waria*:

*“…*. *I feel comfortable and like to access the services at Margaret community health centre*. *The services they [nurses and doctors] provide are very good*. *They are friendly and treat me nicely”*

*(R27*: *47 years old).*

Doctors and nurses at Sugiono [pseudo name] hospital and Margaret community health centre support us [waria community] to access the [HIV/AIDS] services. Even one of the doctors said to us: ‘if you come to control or consult or access ARV, just directly come to my room, you don’t need to queue or wait’

*(R23*: *57 years old).*

*“…*. *when I go to the hospital to do medical check-up*, *the nurses often send me directly to the doctor*, *I don’t have do the registration and wait”*

*(R9*: *32 years old).*

The simplicity of the procedure to access the available services was also identified to be important supporting factor for the accessibility of the services among the study participants. *Waria* populations seemed to be well supported by health professionals including nurses and doctors, and were facilitated to access the services or adhere to medication. The procedure to access the services from them was made simpler than going through the usual hospital or community health centre procedure where registration and waiting for turn would be needed:

*“If we access health services at the [Margaret] community health centre*, *the procedure is not the same as the procedure for general patients*, *it is very quick*. *I just need to say LKB [continuous comprehensive service] data*, *the nurses understand that*. *Sometimes I take medical record sheet and fill it out by myself and then directly go to the doctor’s room”*

*(R5*: *39 years old).*

*“…*. *The procedure is made easier for us*. *This may be because we often have meetings with them [the doctors] and they understand our health conditions and support us*, *we are also very open to them”*

*(R23*: *57 years old).*

### Approachability of HIV-related health services

The existing HIV-related health services seemed to be approachable or known to the study participants through several ways. For example, workshops and regular focus group discussions carried out by health professionals in collaboration with NGOs were mentioned in the interviews as the activities through which information about HIV/AIDS and its related healthcare services were delivered. Through the HIV/AIDS information sessions, *waria* also had the opportunity to raise their concerns in relation to the access to the services. Examples supportive of these statements are:

*“I know about HIV/AIDS and the health services through HIV/AIDS information sessions held by health professionals from community health centres and hospitals in collaboration with the NGO of the waria coordinator*. *I feel like these sessions are very helpful and do encourage me to access the services”*

*(R17*: *44 years old).*

*“We [waria] have regular focus group discussion every month with health professionals from community health centres or hospitals*, *from them I know about HIV/AIDS and HIV/AIDS-related health services and how to access them”*

(R22; 44 years old).

Some participants acknowledged that the information sessions were important motivation that helped them to be healthy. The acquired knowledge from these sessions was instrumental driver enabling them to access HIV/AIDS-related health services:

*“I am lucky because I am regularly involved in activities held by waria friends and an NGO here*, *I got accurate information about treatment and transmission of HIV*, *and I am motivated to become healthy*. *I feel that this has been very much helpful and supports for my treatment”*

*(R12*: *50 years old).*

### Health professionals’ approachability and impact of their positive attitudes

Health professionals’ (doctors and nurses) positive attitudes in delivering HIV/AIDS-related health services at healthcare facilities (both community health centre and hospital) and their ability to encourage the participants to access the services enabled the participants to feel motivated and continue to access the services:

*“…*. *Nurses and doctors are very nice to me*. *I was examined by doctor Nicole [pseudo name] when I was admitted to ICU [Intensive Care Unit]*, *she was very nice to me*. *I feel that it is very easy to access health services like ARV or other health services because nurses and doctors are very helpful and support us [waria]”*

*(R3*: *41 years old).*

*“The health professionals*: *nurses and doctors are very friendly and supportive*. *For example*, *the ones at the Margaret community health centre*. *…*. *The doctors at Sugiono hospital are also very friendly with us [waria]*, *and one of them handles the health problem of waria community here so if we have any difficulties then we can just contact her”*

*(R29*: *36 years old).*

Participants asserted that there was a longstanding reciprocal relationship between their *waria* coordinator and HIV-related health service providers both at the community centres and at the hospitals. The longstanding mutually relationship between the service providers and service receivers seemed to be beneficial and additional enabler to *waria* accessing the services. The following excerpts from interviewees narratives support these assertions:

*“The [waria] coordinator and the doctors and nurses know each other very well*, *so sometimes if I couldn’t go to the hospital [Sugiono] or community health centre [Margaret] to take ARV*, *for instance*, *then the coordinator can make a call and send someone else [a waria friend] to take it*. *I feel that the good relationship between us and the health professional supports our access to services here”*

*(R25*: *43 years old).*

*“They [doctors and nurses] know that we are waria*. *A few doctors are very close to us and we have good relationship with them as we often have meetings with them*. *I get massive support from them to continue my medication …*.”

*(R9*: *32 years old).*

### Affordability of services and proximity to healthcare facilities

Free access to health services including HIV/AIDS-related health services was found to play a positive role in enabling the access to the services. All the interviewees declared to afford the services as they hold health insurance: either Indonesian health card or community health insurance provided by the government of Indonesia. Access to health insurance enhanced *waria’s* affordability in accessing health services especially HIV/AIDS-related health services:

*“Luckily the health services are free of charges because I hold Kartu Indonesia Sehat [Indonesian Health Card]*. *This is very supportive and helpful*. *I feel that the access to HIV/AIDS health services is much easier now because of this”*

*(R8*: *31 years old).*

*“…*. *HIV test is free of charge and ARV is also free*. *This is very helpful*, *and I am still on medication up to now*

*(R13*: *47 years old).*

All the interviewees commented that they lived in the city of Yogyakarta and close to healthcare facilities such as community health centres and hospitals where HIV/AIDS-related health services were available. As such, proximity to HIV/AIDS-related health services was helpful in reaching the healthcare facilities and minimising the transportation cost:

*“I live here [a shelter that belongs to a waria NGO]*. *It is very close to healthcare facilities*. *For instance*, *it takes only a few minutes from here to Margaret community health centre with motorbike taxi [Ojek] and costs me just a few thousand rupiahs every time I go there”*

*(R28*: *30 years old).*

*“I routinely take my medicine [ARV] from the hospital [Sugiono hospital]*. *It is very close from here [participant’s house]*, *I can just walk if I want to or use public transportation*

*(R20*: *37 years old).*

Some participants perceived the need to seek health services, took action and moved to the city of *Yogyakarta* to reach and engage with facilities such as community health centres and hospitals where HIV/AIDS-related health services were easily available and affordable. The following narrations from some participants confirm these views:

*“I have moved here [Yogyakarta] for several years and I found it very helpful*. *It is not far from here [the participant’s house] to hospitals or community health centres*. *I am thankful because I feel that living here which is close to healthcare facilities helps me to regularly do medical check-up and better take care of my health*.”

*(R1*: *55 years old).*

*“Living here [the city of Yogyakarta] makes it easier for me to do the treatment*. *Healthcare facilities are close*. *At least I don’t have to spend much money on transport compared to when I was in my place of origin*. *…*.”

*(R7*: *45 years old).*

## Discussion

The study explored the perceptions of *waria* living with HIV on health system-related factors enabling their access to HIV/AIDS-related health services. The current study reports that HIV/AIDS-related health services were made available at community health centres and hospitals for the *waria* populations and other people living with HIV in Yogyakarta. Supportive of previous studies elsewhere [11, 26, 39], the availability of HIV/AIDS-related services was an instrumental to healthcare service access and utilisation by *waria* populations in Yogyakarta. This study also informs that the participants who acknowledged their needs accessed the available services, including HIV counselling, viral load and CD4 check, STIs, blood sugar, uric acid and cholesterol test, and antiretroviral (ARV) and nutritional foods on regular basis. Consistent with the health care access framework [36], these findings support the notion that, for services to be accessed by the target population, they must be available in the first instance, and appropriate for the intended target population to impart the intended health benefits.

Although the five dimensions offered by Healthcare access framework (approachability, acceptability, availability, affordability, and appropriateness) [36] seemed to be met by the provision of HIV/AIDS related services for *waria* in Yogyakarta, it should be noted that these services were only available in a few community health centres and hospitals in the study setting, reflecting unequal distribution of health resources required for HIV/AIDS health service delivery for *waria* populations as well as other population groups within communities in Yogyakarta and the other parts of Indonesia. Inequity in health service availability has been noted as one of the major barriers to the access to health services by *waria* and other population groups in Indonesia [4, 9, 40–42]. It is also worth noting the current study’s participants’ characteristics that match the five corresponding abilities of the Healthcare access framework (ability to perceive, ability to seek, ability to reach, ability to pay (through possession of health insurances) and ability to engage) (36), resulting into what appeared to be seamless interactions between them and service providers. *Waria* who had either Indonesian Health Card or Community Health Insurance provided by the government were entitled to free access to healthcare services. Likewise, proximity to healthcare facilities seemed to facilitate the participants’ access to the health services available for them. These meant that unaffordability of medical cost and transportation cost to healthcare services which have often been reported in previous studies [43–46] as barriers to healthcare utilisation among population groups within and between societies were not the case among the participants of this study. The current findings indicate that accessibility of HIV/AIDS-related health services are determined by both the five accessibility dimensions and the five corresponding abilities of people. This means that to increase access to such services, healthcare providers need to provide approachable, affordable and appropriate health services that meet the health needs of people living with HIV and to enable them. These findings also echo the principles of Primary Health Care Practice contained in the 1978 Alma Ata Declaration which advocate for services to be accessible, affordable, appropriate and available [47].

Additionally, the study findings suggest that the provision of HIV/AIDS-related health services in this setting supported the Ottawa Charter for Health promotion framework through the creation of supportive environment to attract *waria* to the services, developing personal skills through provision of information to raise awareness of the condition as well as the availability of the services, orienting of the services to target *waria* and other vulnerable populations [48]. These results are also supportive of the findings of previous studies [11, 22–24, 49] indicating knowledge or information about the existence of HIV/AIDS-related health services as an important facilitator of the accessibility to the services by men who have sex with men, sex workers and their clients, transgender persons, students, and general population. In addition, the provision of information would have improved participants HIV-related health literacy. Health literacy is required in making critical health decisions to manage individuals’ health, including to seek and access appropriate health care [36, 50]. To enable smooth interactions between groups and to build trusting partnerships, these activities were reported to be held collaboratively by health professionals from community health centres and hospitals in Yogyakarta and NGOs providing supports for *waria* populations and HIV/AIDS positive people. This is consistent with the findings of previous studies demonstrating positive associations between health information dissemination and access to the health services [10, 27, 51].

In contrast to the findings of previous studies [26, 52, 53] reporting discriminatory attitudes including blatant verbal abuse of healthcare staff against transgender persons and men who have sex with men as significant barriers to their access to HIV/AIDS-related health services, the present study reports experiences of good treatments and positive supports from nurses and doctors who provided the services in both community health centres and hospitals as facilitators of *waria’*s access to HIV/AIDS-related health services at healthcare facilities [54]. Convenience, simplicity of procedure to accessing the HIV/AIDS services as well as the comfort felt when accessing the services at healthcare facilities were also additional data provided by this study as supporting factors for the participants’ access to the health services. Such supports for the participants to continue accessing the health services available for them seem to be a positive consequence of the constructive social relationships between *waria* participants and health professionals in the healthcare facilities in the study setting. This is consistent with the results of previous studies [55–58] indicating positive doctor-patient relationships or interactions as a key factor supportive of healthcare service utilisation among different population groups.

### Reflexivity of the researchers

It is important to acknowledge researchers’ role in the process of knowledge generation to account for biases, personal interpretations or experiences, and to create a balance between personal understanding and participants’ views, and to improve the trustworthiness of the findings [59]. For the current study, all the researchers have strong backgrounds in public health (the senior research is a public health physician) practice and research. They have research experiences in areas of HIV, health services and qualitative methods. They are also strong advocates of the provision of equitable health services, including addressing inequity in access of HIV-related health services for vulnerable populations such as the current study’s participants. It is acknowledged that researchers’ position and background can affect the topic of investigation, the methodology and interpretations of the research findings [60]. Given the experience and background of the current study researchers, it is believed that the research question drove the methodology and methods employed to answer the research question. For example, prior to designing the current study, it was known to researchers that transgender women are a hard to reach population based on factors, such as HIV stigma and stigma of being transgender women. As such, it was known that finding the focal person (i.e. The Director of NGO catering for *waria*) would help in identifying the initial participants who would then help to snowball the recruitment process. Due to the possibility of having only a small number of participants, a qualitative rather than quantitative design was selected for the methodology, and one-on-one interview design was used to collect data and to allow for confidentiality and privacy of participants. A qualitative research was also instrumental in obtaining rich personal narratives of participants in detail. As has been acknowledged, the snowball recruitment strategy led to selecting participants from known networks of initial participants which may have led to preclusion of other potential participants, for example, transgender women who were unknown to current participants. However, as is the case for many qualitative studies, the findings of the current study cannot be generalised to all *waria* populations, but provide rich and detailed information that can be used as evidence for the current research and similar populations. The current study limitations have been acknowledged below.

### Study limitations

The results of this study should be interpreted with caution due to several limitations. First of all, the inclusion criteria for recruitment which required the participants had accessed HIV/AIDS-related health services might have been a limitation as it might have resulted in the neglection of the perceptions of *waria* populations who had not accessed the services on the health system support for the access to HIV/AIDS-related health services. However, as the purpose of this study was to obtain the perceptions of *waria* in Yogyakarta about factors that supported their access to HIV/AIDS-related health services, the insights gained from this study are relevant to informing health interventions that promote access to HIV/AIDS treatment among people living with HIV in general and *waria* populations in particular. Besides, participant recruitment technique that was used might also be a limitation of this study as it might have led to under sampling *waria* outside of the social network of the current participants. Participants in this study were 30 years and older, thus the current findings may be less relevant for younger transgender women. These limitations might have resulted in incomplete overview of the perceptions of *waria* populations on health service system support for the access to HIV/AIDS-related health services in the study setting. Despite these limitations, the results of this study can be useful to inform governments and healthcare providers to strengthen health service system that supports the access to HIV/AIDS-related health services and addresses the needs of *waria* populations and other high-risk groups for HIV infections. Evaluation studies on HIV/AIDS-related health service system, which evaluate availability, approachability, affordability and appropriateness aspects of HIV/AIDS-related health services are recommended. Likewise, future studies in the context of Indonesia and other similar settings, that investigate the abilities of *waria* or transgender populations and other populations living with HIV/AIDS to perceive, seek, reach, pay and engage in HIV/AIDS-related health services are also recommended. The findings indicate some aspects that need to be addressed in HIV-related health service delivery in other parts of Indonesia and other settings where access to the services is still difficult for HIV patients. Those aspects include making HIV/AIDS-related services available in healthcare facilities, disseminating information about the services and how to access the services among vulnerable populations and improving service procedures to facilitate access to the services for people who need them.

## Conclusions

This study reports several health service system-related determinants supportive of *waria’*s access to HIV/AIDS-related health services. These include the free availability and ease of accessibility of the services, convenience and simplicity of care delivery. It is therefore reasonable to allude that the availability of HIV-related services in Yogyakarta for the current study’s participants is an indication of effective performance of the health system, which seems to have addressed inequalities of access in these vulnerable populations in this current setting. It is also reasonable to allude that the duality between the service supply side (Yogyakarta health system / HIV related service providers) and the service receiver side (transgender women communities) have converged—whereas the health care providers are actively seeking for *waria* to come to the services and the *waria* have responded by accessing the provided services, factors that seem to have improved their health outcomes.

The comfort felt by participants due to positive attitudes of health professionals at healthcare facilities, approachability of service providers and well dissemination of information related to the services appeared to have enhanced social interactions and relationships between *waria* and health professionals, and improved participants’ access to the services. The findings also indicate the needs for strengthening and extending HIV/AIDS-related health service system in healthcare facilities in Yogyakarta and other parts of the country to support and provide equal access to HIV/AIDS-related health services for people living with HIV/AIDS especially those who are known to be at high-risk groups for HIV, including transgender populations, sex workers and their clients, men who have sex with men, and other population groups.

## Supporting information

S1 Fig COREQ checklist. (DOCX)

S2 Fig Interview guide. (DOCX)
