# Correction: Perceptions among transgender women of factors associated with the access to HIV/AIDS-related health services in Yogyakarta, Indonesia

**DOI:** 10.1371/journal.pone.0223904

**Published:** 2019-10-09

**Authors:** Nelsensius Klau Fauk, Maria Silvia Merry, Sukma Putra, Mitra Andhini Sigilipoe, Rik Crutzen, Lillian Mwanri

There is an error in affiliation 4 for author Sukma Putra. The correct affiliation 4 is: International Business Management Program, Management Department, BINUS Business School Undergraduate Program, Bina Nusantara University, Jakarta, Indonesia.
