# Supplementary material for: Perceptions among transgender women of factors associated with the access to HIV/AIDS-related health services in Yogyakarta, Indonesia
Source: PLoS One. 2019 Aug 15;14(8):e0221013. doi: 10.1371/journal.pone.0221013 (PMC6695113; doi:10.1371/journal.pone.0221013)
Supplement: S2 Fig — (DOCX) [file pone.0221013.s002.docx]

**PANDUAN WAWANCARA DENGAN WARIA MENGENAI LAYANAN KESEHATAN TERKAIT HIV/AIDS DAN AKSES MEREKA TERHADAP LAYANAN TERSEBUT**

Tanggal : ____________________________(Tanggal)

Pewawancara : ___________________ (Nama)

Partisipan : ________(kode);

Posisi partisipan :___________ (Posisi)

Apakah boleh jika saya merekam wawancara dan membuat catatan selama wawancara?

Apakah Anda ingin seseorang menemani Anda selama wawancara?

Ketika saya menulis hasil penelitian ini, saya akan menggunakan huruf dan nomor identifikasi khusus untuk Anda (serta untuk setiap peserta yang saya wawancarai) untuk memastikan bahwa informasi atau komentar yang Anda berikan dalam wawancara ini tetap anonim. Apakah Anda setuju dengan ini? Anda dapat menarik partisipasi Anda atau meminta saya untuk menghentikan wawancara ini jika Anda tidak ingin melanjutkan atau merasa tidak nyaman selama wawancara ini tanpa konsekuensi apa pun.

Seperti yang Anda ketahui melalui Lembaran Informasi penelitian dan percakapan awal kita ketika Anda mengkonfirmasi kesediaan Anda untuk berpartisipasi, tujuan dari penelitian ini adalah untuk menggali pandangan Anda tentang layanan kesehatan terkait HIV/AIDS yang tersedia di Yogyakarta dan akses Anda terhadap layanan tersebut.

**1. Informasi demografis**

Berapa usia Anda? ___________(tahun)

Kapan Anda terdiagnosa dengan HIV? ___________

Apa tingkat Pendidikan terakhir Anda? Tidak sekolah/SD/SMP/SMA/S1

Apakah Anda perndah terdiagnosa dengan IMS lainnya? ____________

Apakah ada anggota keluarga yang terinfeksi HIV juga? ____________

Apa pekerjaan Anda? ____________

2. Ketersediaan layanan kesehatan terkait HIV/AIDS

Apa yang Anda ketahui tentang layanan kesehatan terkait HIV / AIDS di sini (di Yogyakarta)?

- Apa jenis layanan kesehatan terkait HIV / AIDS yang tersedia?
- Di fasilitas kesehatan apa saja layanan kesehatan terkait HIV tersedia?

3. Kemudahan mengakses dan kemampuan untuk memahami and menjangkau

Bagaimana Anda tahu tentang ketersediaan layanan kesehatan terkait HIV/AIDS?

- Bagaimana Anda mempelajari lebih lanjut tentang masalah HIV/AIDS? Tolong ceritakan.

Apa pendapat Anda tentang penyebaran informasi tentang layanan kesehatan terkait HIV di sini?

Bisakah Anda ceritakan mengenai akses Anda terhadap layanan kesehatan terkait HIV/AIDS yang tersedia di Yogyakarta?

- Seberapa sering Anda mengakses layanan kesehatan terkait HIV / AIDS?
  - Jarang / teratur / bulanan? Mengapa?
- Apa jenis layanan kesehatan terkait HIV / AIDS yang telah Anda akses?

Bisakah Anda berikan gambaran pengalaman Anda pelayanan kesehatan terkait HIV/AIDS yang Anda akses?

- Apakah layanan tersebut mudah/susah diakses? Mengapa?
- Bagaimana Anda dilayani pada saat mengkasesnya? Tolong certitakan.
- Apa yang Anda rasakan ketika mengaksesnya? Bisa ceritakan lebih lanjut.

Bisakah Anda ceritakan mengenai bagaimana Anda menjangkau fasilitas kesehatan yang menyediakan layanan kesehatan terkait HIV/AIDS?

4. Keterjangkauan dan kemampuan membayar

- Apakah layanan terkait HIV / AIDS gratis atau tidak?
- Berapa banyak uang yang Anda habiskan untuk layanan kesehatan terkait HIV / AIDS dan transportasi setiap kali Anda mengaksesnya?
  - Apa pendapat Anda tentang biaya-biaya ini?
    - Apakah Anda pikir Anda mampu membayar biayanya? Ceritakan lebih lanjut tentang itu.
    - Sumber daya apa yang Anda pakai untuk biaya tersebut?
  - Bagaimana Anda mengatasi pengeluaran/biaya kesehatan dan transportasi?

5. Kesesuaian dan kemampuan untuk terlibat/mengambil bagian dalam layanan kesehatan

Apakah menurut Anda layanan kesehatan terkait HIV / AIDS yang tersedia di sini sesuai atau yang Anda butuhkan? Jelaskan tentang itu.

Apakah menurut Anda layanan diberikan dengan cara yang baik oleh penyedia layanan kesehatan yang berkualitas? Tolong ceritakan lebih lanjut tentang ini.

Apa yang memotivasi Anda untuk mengakses layanan?

6. Penerimaan dan kemampuan untuk mencari

Bagaimana prosedur untuk mengakses layanan kesehatan terkait HIV / AIDS?

- Apa pendapat Anda tentang prosedur tersebut? (Mendukung atau menghambat?) Ceritakan tentang hal itu.

Siapa penyedia layanan kesehatan terkait HIV yang melayani Anda?

- Dokter/perawat?
- Apakah Anda yang Anda rasakan mengenai pelayanan yang mereka berikan? Tolong ceritakan lebih lanjut.
  - Sikap dan prrilaku mereka ketika melayani Anda
  - Apakah Anda merasa mendapat dukungan atau tidak dari dokter atau perawat yang melayani Anda? Tolong ceritakan lebih lanjut tentang hal ini?
  - Apakah ini mempunya pengaruh terhadap Anda dalam mencari dan mengakses layanan kesehatan? Tolong ceritakan.

Bisakah Anda ceritakan mengenai relasi Anda sosial Anda sebagai pasien dengan dokter atau perawat yang memberikan pelyanan terkait HIV/AIDS?

- Apakah kalian saling mengenal satu sama lain dengan baik?
- Apaka hubungan sosial dengan para petugas kesehatan mempunyai pengaruh terhadap Anda dalam mencari atau mengkases layanan kesehatan? Tolong ceritakan lebih lanjut.

Apakah ada hal lain yang ingin Anda tambahkan?

Apakah Anda ingin melihat salinan transkrip wawancara dan mengeditnya sebelum saya menganalisis informasi?

Apakah Anda bisa menyarankan Waria lain yang mungkin bersedia berpartisipasi dalam penelitian ini?

**INTERVIEW GUIDE FOR WARIA ABOUT HIV/AIDS-RELATED HEALTH SERVICES AND THEIR ACCESS TO THE SERVICES**

Date: ____________________________(Date)

Interviewer: ___________________ (Name)

Interviewee: ________(Code);

Position of the interviewee: ___________ (Position)

Is it OK if I record the interview and take notes during the interview?

Would you like to have someone with you while we talk?

When I write up the results of this study, I will assign a specific study identification letter and number for you (as well as for each participant I interview) to ensure that information or comments you provide in this interview remain anonymous. Is this OK with you? You can withdraw your participation or ask me to stop this interview if you do not want to continue or feel uncomfortable during this interview without any consequences.

As you have known through the study information sheet and our initial conversation once you confirmed your willingness to participate, the purpose of this study is to gain your insights about HIV/AIDS-related health services in Yogyakarta and your access to the services.

**1. Demographic information**

How old are you? ___________(in years)

In what year were you diagnosed with HIV? ___________(in years)

What is the highest education you received? Not at all/primary/secondary/tertiary

Have you ever been diagnosed with sexually

transmitted infections other than HIV? ____________

Are the any other family members of

yours who are living with HIV? ____________

What is your occupation? ____________

2. Availability and ability to perceive:

What do you know about HIV/AIDS-related health services here (in Yogyakarta)?

- What types of HIV/AIDS-related health services are available?
- At which healthcare facilities are they available/offered?

3. Approachability and ability to perceive and reach:

How do you know about the availability of HIV/AIDS-related health services? Tell me more about it.

- How do learn about HIV/AIDS?

What do you think about dissemination of information about HIV related health services here?

Would you mind describing about your access to HIV/AIDS-related health services in Yogyakarta?

- How often do you access HIV/AIDS-related health services?
- Rarely/regularly/monthly? Why?
- What kinds of HIV/AIDS-related health services have you accessed?

Would mind sharing your experience about HIV/AIDS-related health services you have accessed?

- Were the services easy/difficult to access? Please explain.
- How were you served by health professionals? Tell me more about it.
- How did you feel about the services?

How do you get to any of the health facilities to access HIV/AIDS-related health services?

4. Affordability and ability to pay

- Are HIV/AIDS-related services free or paid?
- How much money do you spend on HIV/AIDS services and transport every time you access them?
- What do you think about these costs?
  - Affordable?
  - What sorts of resources do you spend on the costs?
- How do you cope with transport and health expenditures?

5. Appropriateness and ability to engage

Do you think the HIV/AIDS-related health services available here are appropriate or the ones that you need? Explain about it.

Do you think the services are delivered in a good way by qualified health service providers? Please tell me more about this.

What motivates you to access the services?

6. Acceptability and ability to seek

What are the procedures to access HIV/AIDS-related health services?

- What do you think about the procedures? (Supportive or inhibitive?) Tell me about it.

Who are the HIV-related health service providers that serve you?

- Doctors/nurses?
- What do you feel about services they provide? Tell me more about these.
  - The attitude and behaviours once providing the services
  - Do you feel you get support from doctors and nurses who serve you? Tell me more about it.
  - Do these have an influence on you in seeking HIV services?

Would you describe your social relationship as a patient with doctors or nurses who serve you?

- Do you know each other?
- Does your social relationship with health professionals have an influence on you in seeking or accessing health services? Tell me more about it.

Is there anything else you wish to add?

Would you like to see a copy of the interview transcript and edit it prior to me analysing the information?

Would you be able to suggest other transgender women who might be willing to participate in this study?
